# Etiology of Frontal Network Syndromes in Isolated Subtentorial Stroke

**DOI:** 10.3233/BEN-2008-0220

**Published:** 2009-07-29

**Authors:** Michael Hoffmann, Lourdes Benes Cases

**Affiliations:** Cognitive Neurology DivisionDepartment of NeurologyUniversity of South FloridaTampaFL 33612USA

## Abstract

*Background:* The neurobiology of the frontal network syndrome (FNS) that may occur with isolated subtentorial stroke is unknown.

*Aim:* Evaluate for frontal network syndromes in young people post subtentorial stroke who have recovered neurologically and compare to a stroke lesion group least likely to manifest frontal network syndromes.

*Methods:* Young people (18–49 years) with isolated cerebellar or brainstem subtentorial stroke (ST) that had recovered to independency (Rankin score ࣘ 2) with minimal or no residual neurological deficit (NIHSS ࣘ 4) with neurological recovery enabling resumption of former employment. Comparison was made to age and education matched young people with posterior circulation territory parieto occipital lobe infarcts (PO). Depression, anxiety, systemic disease, autoimmune disease, neurodegenerative disease and substance abuse were specific exclusions. A battery of frontal tests surveying the principal frontal network syndromes (apathy, disinhibition, executive dysfunction, emotional intelligence quotient) was used. Neurological deficit and long tract signs were measured by the NIH stroke score (NIHSS).

*Results:* From the cognitive stroke registry of young stroke patients (*n* = 511), analysis for isolated subtentorial infarction yielded cerebellar infarcts (*n* = 43, 8.4%) and brainstem infarcts (*n* = 36, 7.0%). After exclusions, 16 patients (cerebellum, *n* = 10, pons, *n* = 6) were compared to 10 PO infarct patients controlled for mean age, gender and NIH stroke scores. Overall 11/16 (69%) patients in the ST and 5/10 (50%) in the PO group manifested one or more of the principal FNS syndromes. Mean T scores for apathy, disinhibition, executive function and emotional intelligence standard scores were significantly more impaired in the ST group, but not for WCST error percentage scores.

*Conclusions:* The mismatch of scant neurological deficit manifested by low NIHSS but with FNS in the majority of isolated ST stroke and more so than with PO stroke, gives support for a state dependent or neurotransmitter perturbation. The clinical impact is that such syndromes may be amenable to neuropharmacological intervention.

## 1. Introduction

The neurobiology of cognitive impairment in isolated subtentorial stroke is unknown. Frontal network syndromes have been reported with isolated brainstem stroke with lesions in midbrain, pontine and even medullary locations [1–11]. Likewise, the cognitive impairment that has been reported with isolated cerebellar strokes is mainly a dysexecutive syndrome [12–14]. We sought to determine whether the lesion effects of isolated subtentorial stroke suggested dysfunction primarily in the state dependent aminergic systems (as opposed to a channel dependent process) by investigating patients that had recovered neurologically and compared to a stroke lesion group least likely to manifest frontal network syndromes.

## 2. Methods

### 2.1. Recruitment

Consecutive stroke patients were accrued through a prospectively coded dedicated cognitive stroke registry, as part of a tertiary care JCAHO primary and Comprehensive Stroke Center. All patients were examined and

[1]Presented in part at the 18th Annual American Neuropsychiatric Association Meeting in Tucson Arizona, February 17–20, 2007.

ISSN 0953-4180/08/$17.00 © 2008 – IOS Press and the authors. All rights reserved managed by board certified neurologists. The Stroke registry was approved by the University Institutional Review Board and in compliance with HIPAA regulations. All patients signed informed consent for the evaluation and the collection of the their neurological, medical and neurocognitive data. Young people (18–49 years) with stroke, were tested with a bedside cognitive screening examination and pending the results, tested further with neuropsychological metric tests within one month of stroke onset. Those patients with isolated cerebellar or brainstem subtentorial stroke (ST) that had recovered to independency (Rankin score $\leqslant 2$) with minimal or no residual neurological deficit (NIHSS $\leqslant$ 4), enabling resumption of former employment were selected and tested with a more extensive battery.

### 2.2. Inclusion and exclusion criteria

Only patients with isolated cerebellar stroke or isolated brainstem stroke were evaluated. To obviate confounding comorbid conditions, a comprehensive list of exclusions were applied. 1. Cerebrovascular exclusions: concomitant supratentorial cortical infarcts, concomitant subcortical infarcts, leukoaraiosis. 2. Other neurological exclusions included: moderate or severe aphasia, encephalopathy, hydrocephalus, substance abuse, infective or metabolic processes, Alzheimer's disease, other dementias, inability to complete cognitive testing or moderate severe neurological deficit (NIHSS score $> 4$ or Rankin $> 2$). 3. Neuropsychiatric exclusions included: moderate or severe depression, anxiety and psychosis based on DSM-IV criteria [15] because of the effect on cognitive metric testing.

### 2.3. Neuropsychological procedures

The Boston naming Test was administered to screen for significant aphasia [16]. A battery of frontal tests surveying the principal frontal network syndromes of apathy, disinhibition and executive dysfunction were administered [17]. In addition newer measures that measure emotional intelligence quotient [18] were employed in addition to the revered Wisconsin Card Sorting Test (WCST) [19]. Depression was assessed by the Carroll Depression Scale [20] and moderate to severely depressed patients were excluded from further analysis. Neurological deficit and long tract signs were measured by the NIHSS [21]. A uniform pathophysiological entity of only bland cerebral infarction was investigated. Comparison was made to age, gender, education matched and NIHS score admission deficit young people with posterior circulation territory parieto occipital lobe infarcts (PO). Lesion topography was determined by the digitized clinical brain atlas [22].

### 2.4. Stroke protocol

All patients had a standardized stroke protocol evaluation incorporating complete blood, count, electrolytes, blood urea nitrogen, creatinine, lipid panel, homocysteine, C – reactive protein, chest radiograph, electrocardiogram, multimodality (GE 1.5 Tesla) MRI (T1, T2), fluid attenuation inversion recovery (FLAIR), diffusion weighted imaging (DWI) magnetic resonance angiography (MRA) (intracranial and cervicocephalic), echocardiography (transthoracic or transesophageal) and duplex Doppler sonography. Standardized qualitative stroke scores included the NIHSS and Rankin scores.

### 2.5. Stroke severity and etiology

Lesion severity was graded with the NIHSS and stroke etiology was evaluated according to the TOAST classification (Trial of Org 10172 in Acute Stroke Trial) by one of the two attending stroke neurologists. This mechanistic classification of stroke includes large vessel disease, small vessel disease, cardioembolic, other and unknown entities [23]. An expanded version of the category "other" was used; cerebral venous thrombosis, vasculitis, prothrombotic disorders, dissection and other vasculopathy such as posterior reversible encephalophatay syndrome (PRES), eclampsia, cerebral vasospasm, dolichoectasia and migraine related stroke.

### 2.6. Neuroimaging

Lesion location and cerebral localization by MRI was performed according to the 3 dimensional co-planar stereotaxic digital human brain atlas, Cerefy Clinical Brain atlas version 2.0 (2004).

### 2.7. Statistical analyses

For numeric data comparisons, with two groups comparison, the t test was used, assuming normal distribution. Analysis of variance (ANOVA) was used for more than two groups comparison with 1 continuous outcome. Multivariate analysis of variance (MANOVA) was used for more than 1 continuous outcome (here we have 6 outcomes). Principal component analysis, a method to compress data was used to depict the composite data in Fig. 1.

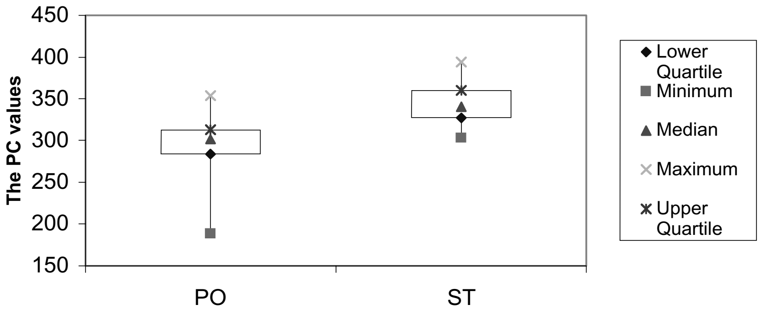

Fig. 1. Principal Component analysis Result. The t test shows difference between the PO and ST groups ($t$ = 4.2, df = 24, $P$ = 0.0003).

## 3. Results

From the cognitive stroke registry of young stroke patients ($n$ = 511), analysis for isolated subtentorial infarction yielded cerebellar infarcts ($n$ = 43, 8.4%) and brainstem infarcts ($n$ = 36, 7.0%). After exclusions, 16 patients (cerebellar hemisphere $n$ = 10, pons, $n$ = 6) were compared to 10 PO infarct patients. In the PO group, 3 were with left hemisphere, 7 with right hemisphere involvement, parietal only ($n$ = 4), parietotemporal ($n$ = 2), parietoccipitotemporal ($n$ = 3) and occipital only ($n$ = 1). In the ST group, there were 3 right and 3 left partial hemipontine infarcts and in the cerebellar group there were 4 right sided, 3 left sided and 3 bilateral hemisphere cerebellar infarcts. The infarct vascular territories were PICA ($n$ = 5), SCA ($n$ = 3), AICA ($n$ = 1) and vermal ($n$ = 1). These patients were controlled for mean age (PO = 50.2 years, ST = 53.1 years, $p$ = 0.5), gender (women numbered; PO 5/10 and ST 7/16) and NIHSS at presentation of stroke (ST = 2.1 95% CI: 0.7–3.4 and PO = 4.0, 95% CI: 1.5–6.5, $p$ = 0.14).

Overall 11/16 (69%) patients in the ST and 5/10 (50%) in the PO group manifested one or more of the principal FNS syndromes. Mean apathy T scores (PO: 52.2, ST: 66.3, $t = -2.3$, $p$ = 0.02), disinhibition T scores (PO: 43.0, ST:63.5, $t = -4.3$, $p$ = 0.003), executive function T scores (PO: 50.0, ST: 66.6, $t = -4.1$, $p$ = 0.004), emotional intelligence standard scores (PO: 113.1, ST: 91.3, $t = -4.05$, $p$ = 0.0007), were all different between the 2 groups, but not for WCST error percentage T score (PO: 52.6, ST: 46.2, $t$ = 1.5, $p$ = 0.13) (Table 1). MANOVA was performed with Wilks Lambda = 0.5289, F value = 3.38, numerator degree freedom-5, denominator degree freedom = 19, p value = 0.0236. See figure 1 for comparative box plots for the ST and PO groups for the 6 frontal network scores. The principal component analysis was created based on all 6 measures and t test revealed a significant difference.

## 4. Discussion

Notwithstanding the large number of stroke patients screened, this small select sample is notable for the dichotomy of cognitive impairment in the context of relatively normal neurological functioning, at least for elementary neurological deficits or long tract signs. Furthermore, these 16 patients were able to resume their former employment but not without subjective difficulty. Contemporary understanding of brain network functioning describes brain function according to hard-wired networks and chemically addressed systems [24]. The former include five large-scale distributed networks (frontal, language, limbic/memory, object/face recognition, spatial orientation). The frontal network in turn is comprised of 5 circuits, 3 neurobehavioral (dorsolateral, orbitofrontal and anterior cingulate) and 2 motor (oculomotor and motor) [25]. The chemically addressed systems or state dependent systems are in turn comprised of five different neurotransmitter systems (serotonin, dopamine, norepinephrine, acetylcholine, histamine) that modulate cerebral networks for faculties such language, attention, memory, spatial orientation, emotion and frontal networks [26–32]. Chemically addressed systems are regarded as providing a matrix that influences the state of information processing. Clinical testing of these frontal or executive syndromes is challenging with both neuropsychological and bedside approaches having merit. Brain lesions may differentially impair these systems [33–37].

With respect to postulated pathophysiological processes, the contralateral cortical diaschisis due to cerebellar lesions is one possible explanation [38,39] but may still reflect a chemical or neurotransmitter related function. The clinical relevance of these findings is that chemically addressed systems are amenable to neuropharmacological therapies with many classes (serotonergics, dopaminergics, cholinomimetics, psychos-

Table 1
Demographics, clinical and cognitive characteristics in the ST and PO groups

| | ST | PO | P value |
|---|---|---|---|
| Demographics | | | |
| Total Number | 16 | 10 | – |
| Gender: women/men | 7/16 | 5/5 | NS |
| Mean Age | 50.2 | 53.1 | 0.5 |
| Stroke Severity | | | |
| NIHSS | 2.1 | 4.0 | 0.14 |
| Stroke Mechanisms (TOAST) | | | |
| Other | 4 | 6 | NS |
| Small vessel disease | 3 | 4 | NS |
| Large vessel disease | 2 | 5 | NS |
| Cardioembolism | 1 | 1 | NA |
| Cognitive Metric | | | |
| Apathy (mean T score) | 66.3 | 52.2 | 0.02 |
| Disinhibition (mean T score) | 63.5 | 43.0 | 0.003 |
| Executive (mean T score) | 66.6 | 50.0 | 0.004 |
| Emotional Intelligence (SS) | 91.3 | 113.1 | 0.0007 |
| Wisconsin Card Sorting Test (T score) | 46.2 | 52.6 | 0.13 |

Legend:
SS: Standard Scores.
NS: Not significant.
NA: Not applicable.

timulants) of drugs currently available. The literature is replete with case series and anecdotes in the treatment of frontal lobe disorders. Some success has been reported with the attentional disorders that accompany stroke with methylphenidate, dextroamphetamine, pemoline and modafanil [40]. Modification of disinhibition behaviors, often seen in patients with orbitofrontal injuries with antipyschotics, benzodiapezines, buspirone, carbamazepine, trazadone, propranolol, valproate, antidepressants and lithium has had some success [41,42]. The apathy accompanying the medial frontal syndrome has been shown to improve anecdotally with psychostimulants or dopamine receptor agonists [43]. Cholinomimetic agents (donepezil, galantamine, rivastigmine) also provide modest improvements in memory as well as other cognitive functions such as psychosis, agitation, apathy, disinhibition and aberrant motor behavior [44,45].

Potential criticisms of this report are undoubtedly the small sample size, which predispose to a type II error. Given the highly select group of only young patients with discreet strokes in the subtentorial regions with good enough recovery to return to employment, this was not surprising. Much larger stroke databases in excess of approximately 10000 patients will be required to improve this sample size and allow more secure statistical comparisons. This report is therefore hypothesis generating in terms of the exploratory rather than strictly significant data presented.

In conclusion, the mismatch of scant neurological deficit manifested by low NIHSS but with FNS in the majority of isolated ST stroke and more so than with PO stroke, gives support for a state dependent or neurotransmitter perturbation. The clinical impact is that such syndromes may be amenable to neuropharmacological intervention.

## Acknowledgment

In part by a NIH K 12 grant from the University of Kentucky, Lexington Kentucky.

## References

[1] M. Van Zandvoort, E. de Haan, J. van Gijn and L.J. Kapelle, Cognitive functioning in patients with a small infarct of the brainstem, *J Int Neuropsychol Soc* **9** (2003), 490–494.
[2] M. Hoffmann and F. Schmitt, Cognitive impairment in isolated subtentorial stroke, *Acta Neurologica Scandinavica* **109** (2004), 14–24.
[3] M. Hoffmann and A. Watts, Cognitive Dysfunction in Isolated Brainstem Stroke. A Neuropsychological and SPECT study, *Journal of Stroke and Cerebrovascular Diseases* **7** (1998), 24–31.
[4] P. Garrard, D. Bradshaw, H.R. Jaeger, A.J. Thompson, N. Losseff and D. Playford, Cognitive dysfunction after isolated brain stem insult. An underdiagnosed cause of long term morbidity, *J Neurol Neurosurg Psychiatry* **73** (2002), 191–194.

[5] E. Goldberg, R.M. Bilder, J.E. Hughes, S.P. Antin and S. Mattis, A reticulo-frontal disconnection syndrome, *Cortex* **25** (1989), 687–695.

[6] J.C. Adair, D.J. Williamson, R.L. Schwartz and K.M. Heilman, Ventral tegmental area injury and frontal lobe disorder, *Neurology* **46** (1996), 842–843.

[7] P. Winn, Frontal syndrome as a consequence of lesions in the pedunculopontine tegmental nucleus: a short theoretical review, *Brain Res Bull* **47** (1998), 551–563.

[8] G. Nelles, K.A. Cotonis and S.L. Valente, Recovery following lateral medullary infarction, *Neurology* **50** (1998), 1418–1422.

[9] D. Evyapan and E. Kumral, Pontine anosognosia for hemiplegia, *Neurology* **53** (1999), 647–664.

[10] B. Crosson and G.M. Tauft, Cortical functioning during recovery from brainstem infarction: a case report, *International Journal of Clinical Neuropsychology* **2** (1981), 3–7.

[11] M. Hoffmann, Higher Cortical Functions After Stroke: An Analysis of 1000 Patients from a Dedicated Cognitive Stroke Registry, *Neurorehabilitation and Neural Repair* **15** (2001), 113–127.

[12] J. Malm, B. Kristensen and T. Karlsson, Cognitive impairment in young adults with infratentorial infarcts, *Neurology* **50** (1998), 1418–1422.

[13] J.D. Schmahmann, An emerging concept. The cerebellar contributions to higher function, *Arch Neurol* **48** (1991), 1178–1187.

[14] J.D. Schmahmann and J.C. Sherman, The cerebellar cognitive affective syndrome, *Brain* **121** (1998), 561–579.

[15] American Psychiatric Association, Diagnostic and statistical manual of mental disorders (4th ed.), Washington, DC: Author, 1994.

[16] E. Kaplan, H. Goodglass and S. Weintraub, Boston Naming Test version 2. Philadelphia: Lea & Febiger, 2001.

[17] J. Grace and P.F. Malloy, *Frontal Systems Behavior Scale*, Lutz Florida 2002, PAR.

[18] R. Baron, Baron Emotional Quotient Inventory, MHS Toronto 1997.

[19] R. Heaton, Wisconsin Card Sorting Test (WCST) computer version 4 Odessa, FL: 2004, Psychological Assessment Resources.

[20] B. Carroll, Carroll Depression Scales, Toronto, MHS Heaton, R. K, PAR staff, 1998.

[21] P. Lyden, T. Brott, B. Tilley, K.M. Welch, E.J. Mascha, S. Levine, E.C. Haley and J. Grotta, Marler, Improved reliability of the NIH Stroke Scale using video training, *NINDS TPA Stroke Study Group J Stroke* **25**(11) (November 1994), 2220–2226.

[22] W.L. Nowinski and A. Thirunavuukarasuu, The Cerefy Clinical Brain Atlas on CD-ROM 2nd edition, Thieme 2004, Stuttgart.

[23] H.P. Adams, B.H. Bendixen, L.J. Kappelle, J. Biller, B.B. Love and D.L. Gordon, Marsh III EE and TOAST investigators. Classification of Subtype of Acute Ischaemic Stroke, *Stroke* **24** (1993), 35–41.

[24] M.-M. Mesulam, Behavioral Neuroanatomy: Large Scale Networks, Association Cortex, Frontal Syndromes, the Limbic System and Hemispheric Specializations. in: *Principles of Behavioral and Cognitive Neurology*, M.-M. Mesulam, Oxford University Press, New York 2000.

[25] T.W. Chow and J.L. Cummings, Frontal-Subcortical Circuits, in: *The Human Frontal Lobes*, B.L. Miller and J.L. Cummings, eds, The Guilford Press, New York, 1999.

[26] E. Boller and H. Spinnler, eds, The Frontal Lobes, in: *Handbook of neuropsychology*, F. Boller and J Grafman, eds, Section 12, Amsterdam, the Netherlands, Elsevier, 1994.

[27] D.R. Britton, C. Ksir, K.T. Britton, D. Young and G.F. Koob, Brain norepinephrine depleting lesions selectively enhance behavioral responsiveness to novelty, *Physiol Behav* **33** (1984), 473–478.

[28] N.R.W. Seldon, T.W. Robbins and B.J. Everitt, Enhanced behavioral conditioning to context and impaired behavioral and neuroendocrine responses to conditioned stimuli following ceruleocortical noradrenergic lesions: support for an attentional hypothesis of central noradrenergic function, *J Neurosci* **10** (1990), 531–539.

[29] W. Schultz, Dopamine neurons and their role in reward mechanisms, *Curr Opin Neurobiol* **7** (1997), 191–197.

[30] P. Boulenguez, N. Foreman, J. Chauveau, L. Segu and M.C. Buhot, Distractibility and locomotor activity in the rat following intracollicular injection of a serotonin 1B-1D agonist, *Behav Brain Res* **67** (1995), 229–239.

[31] H. Wada, N. Inagaki, A. Yamatodani and T. Watanabe, Is the histaminergic neuron system a regulatory center for whole brain activity? *Trends in Neurosci* **14** (1991), 415–418.

[32] P. Simon, Dopaminergic A10 neurons and frontal systems, *J Physiol* (*Paris*) **77** (1981), 81–95.

[33] A. Luria, *Higher Cortical Functions in Man*, New York Basic Books, New York 1966.

[34] T. Ettlin and U. Kischka, Bedside Frontal Lobe Testing, pp 233-246 in: *The Human Frontal Lobes*, B.L. Miller and J.L. Cummings, eds, Guilford Press, New York, 1999.

[35] S.W. Anderson, H. Damasio, R.D. Jones and D. Tranel, Wisconsin Card Sorting Test as a measure of frontal damage, *Journal of Clinical and Experimental Neuropsychology* **13** (1991), 909–922.

[36] G.J. O'Shanick and A.M. O'Shanick, Personality and intellectual changes, in: *Neuropyschiatry of Traumatic Brain Injury*, J.M. Silver, S.C. Yudosfsky and R.E. Hales, eds, Washington DC: American Psychiatric Press 1994, pp. 163–188.

[37] J.M. Silver and S.C. Yudofsky, Pychopharmacology, in: *Neuropyschiatry of Traumatic Brain Injury*, J.M. Silver, S.C. Yudosfsky and R.E. Hales, eds, Washington DC: American Psychiatric Press, 1994, pp. 631–670.

[38] A.B. Newberg, A. Alavi and J. Alavi, Contralateral cortical diaschisis in a patient with cerebellar astrocytoma after radiation therapy, *Clin Nucl Med* **25** (2000), 431–433.

[39] B. Infield, S.M. Davis, M. Lichtenstein, P. Mitchell and J.L. Hooper, Crossed cerebellar diaschisis and brain recovery after stroke, *Stroke* **26** (1995), 90–95.

[40] E.D. Ross and R.M. Stewart, Akinetic mutism from hypothalamic damage: successful treatment with dopamine agonists, *Neurology* **31** (1981), 1435–1439.

[41] J.T. Stewart, M. Leadon and L.J. Gonzalez Rothi, Treatment of a case of akinetic mutism with bromocriptine, *J Neuropsychiatry Clin Neurosci* **2** (1990), 462–463.

[42] R.W. Parks, D.J. Crockett et al., Assessment of bromocriptine intervention for the treatment of frontal lobe syndrome: a case study, *J Neuropsychiatry Clin Neurosci* **4** (1992), 109–111.

[43] R.S. Marin, B.S. Fogel et al., Apathy: a treatable syndrome, *J Neuropsychiatry Clin Neurosci* **7** (1995), 23–30.

[44] J.L. Cummings, Cholinesterase inhibitors: a new class of psychoactive agents, *Am J Psychiatry* **157** (2000), 4–15,

[45] D.I. Kaufer, J.L. Cummings and D. Christine, Effecrts of tacrine on behavioral symptoms in Alzheimer's disease: An open label study, *J Geriatric Psychiatry Neurol* **9** (1996), 1–6.
